# Evaluation of Intrusion Detection Systems

**DOI:** 10.6028/jres.108.040

**Published:** 2003-12-01

**Authors:** Jacob W. Ulvila, John E. Gaffney

**Affiliations:** Decision Science Associates, Inc., Vienna, VA 22818; Lockheed Martin, Gaithersburg, MD 20879

**Keywords:** Bayesian statistics, computer security, decision analysis, intrusion detection, receiver operating characteristic (ROC), software evaluation

## Abstract

This paper presents a comprehensive method for evaluating intrusion detection systems (IDSs). It integrates and extends ROC (receiver operating characteristic) and cost analysis methods to provide an expected cost metric. Results are given for determining the optimal operation of an IDS based on this expected cost metric. Results are given for the operation of a single IDS and for a combination of two IDSs. The method is illustrated for: 1) determining the best operating point for a single and double IDS based on the costs of mistakes and the hostility of the operating environment as represented in the prior probability of intrusion and 2) evaluating single and double IDSs on the basis of expected cost. A method is also described for representing a compound IDS as an equivalent single IDS. Results are presented from the point of view of a system administrator, but they apply equally to designers of IDSs.

## 1. Introduction

Little was done to evaluate computer intrusion detection systems (IDSs) prior to the evaluations conducted by the Massachusetts Institute of Technology’s Lincoln Laboratory under the sponsorship of the DARPA in 1998. This effort is known as the 1998 DARPA off-line intrusion detection evaluation. It was the first comprehensive test of multiple IDSs using a realistic setting. Various accounts of this evaluation have been published by Durst et al. [1], McHugh [2], Lippmann et al. [3], Stolfo et al. [4], and McHugh et al. [5]. This evaluation was the first that evaluated many IDSs, used a wide variety of intrusions, simulated realistic normal activity, and produced results that could be shared by many researchers.

During the 1998 DARPA evaluation, detection results were combined with the total number of network sessions to give two summary measures of an IDS’s performance: detection rate (intrusions detected divided by intrusions attempted) and false alarm rate (false alarms divided by total network sessions). These summary measures were taken as an estimate of one point on the IDS’s receiver operating characteristic (ROC) curve. A ROC curve is a plot of detection probability versus false alarm probability. It shows the probability of detection provided by the IDS at a given false alarm probability. Alternatively, it shows the false alarm probability provided by the IDS at a given probability of detection.

Lippmann et al. [3] claim, “a novel feature of this evaluation is the use of receiver operating characteristic (ROC) techniques to evaluate intrusion detection systems.” Although Lippmann et al. [3] used ROC curves, their evaluations were based on simply comparing ROC curves for dominance. A dominant curve would lie above and to the left of a dominated curve. No metric was presented for the degree of dominance, nor was any statement made as to the value of one IDS over another or the value of an IDS over no IDS. Others, however, have proposed metrics for evaluating the ROC curves of IDSs. Durst et al. [1] contend that, “the area under the curve is one measure of an intrusion detection system’s effectiveness.” Axelsson [6] proposes a “required level of false alarms;” Durst et al. [1] suggest a false alarm rate that is “manageable.” Saydjari [7] proposes a goal on detection probability and probability of false alarm. Presumably, metrics could be developed (e.g., Euclidean distance) that describe how “close” a given ROC curve is to the required level or goal. However none of these metrics is satisfactory in that none provides a complete measure of the capability of an IDS.

Stolfo et al. [4] propose an alternative method for evaluating IDSs that is based on cost metrics. They claim to, “demonstrate that the traditional statistical metrics used to train and evaluate the performance of learning systems (i.e., statistical accuracy or ROC analysis) are misleading and perhaps inappropriate for this application.” They claim that their cost-based metrics are more appropriate, and they further, “demonstrate how the [cost-based] techniques developed for fraud detection can be generalized and applied to the important area of intrusion detection.” They apply their cost-based methods by calculating the total costs incurred with different IDSs by adding the costs from a number of simulation trials. They do not show how their method uses all of the information in a ROC curve, nor do they provide a compelling demonstration of the superiority of the cost metric.

We demonstrate that both the ROC analysis and other cost analysis methods that we have reviewed are incomplete. Furthermore, we demonstrate how a decision tree can combine and extend the ROC and cost analysis methods to provide an expected cost metric that reflects the intrusion detection system’s ROC curve, cost metrics, and an assessment of the hostility of the environment as summarized in the prior probability of intrusion. We further demonstrate how this method can be used to: decide the optimal operating point on an IDS’s ROC curve, choose the best intrusion detection system, determine the value of one intrusion detection system over another, determine the value of an IDS over no IDS, and determine how to adjust the operation of an IDS to respond to changes in its environment.

McHugh’s [2] very thorough critique of the 1998 DARPA evaluation raises a number of serious questions about how the ROC curves in it were constructed. He also raises concerns about the appropriateness of ROC analyses for these evaluations at all, especially if the unit of measurement is different for different IDSs. We do not address how the ROC curves are obtained; we show how they should be compared once they have been obtained.

This paper is arranged as follows. Section 2 describes our method for evaluating a single IDS. It describes ROC curves, presents a decision tree analysis for determining an IDS’s optimal operating point, and shows how the expected cost of operating an IDS in a hostile environment can be used to evaluate an IDS. Section 2 also describes a method for determining the expected value of one IDS over another. We demonstrate that this expected value depends on the costs of mistakes, the probability of intrusion, and the IDSs’ ROC curves, not just some of these factors. We demonstrate that the area under a ROC curve is not a valid measure of an IDS’s effectiveness, contrary to the assertions of Durst et al. [1].

Section 3 extends the method to evaluate a compound IDS that consists of two independent IDSs. Results are presented that describe the optimal operation of the combination of two IDSs and compare the expected cost from a single IDS with that from a compound IDS. Results are shown for a compound IDS composed of two independent identical IDSs, two independent different IDSs, and two independent IDSs, one with a zero probability of false alarms.

Section 4 describes how a compound IDS can be represented by a single, composite ROC curve that is derived from the ROC curves of its components.

Section 5 presents conclusions, recommendations, and suggested extensions of the method.

Four appendices contain technical details. Appendix A (Sec. 6) shows the analysis for a compound IDS with a single decision. Appendix B (Sec. 7) shows the analysis for a compound IDS with sequential decisions. These appendices show that the expected cost from using a compound IDS composed of two independent IDSs is the same regardless of whether the response decision is made sequentially after each component IDS’s report or if the response decision is made only once on the basis of both reports. Appendix C (Sec. 8) shows simplified analyses and the geometry of the ROC. It describes the conditions under which the embedded decision can be removed from the decision tree, describes an analysis of the ROC convex hull, and describes an extension of the analysis that includes additional costs. Appendix D (Sec. 9) shows the derivation of a single, composite ROC curve to represent the performance of multiple IDSs.

## 2. Evaluation of a Single Intrusion Detection System (IDS)

A computer intrusion detection system (IDS) is concerned with recognizing whether an intrusion is being attempted into a computer system. An IDS provides some type of alarm to indicate its assertion that an intrusion is present. The alarm may be correct or incorrect. A decision maker (e.g., the system administrator) can decide to respond to the alarm or to ignore the alarm. This section describes a decision analysis method for determining the best operating point for an IDS and an expected cost metric that can be used to evaluate an IDS.

An IDS’s receiver operating characteristic (ROC) curve describes the relationship between the two operating parameters of the IDS, its probability of detection, 1−*β*, and its false alarm probability, *α*. That is, the ROC curve displays the 1−*β* provided by the IDS at a given *α*. It also displays the *α* provided by the IDS at a given 1−*β*. The ROC curve thus summarizes the performance of the IDS. We do not address how one generates this ROC curve, just what to do with it after it is determined.

**Fig. 1:**
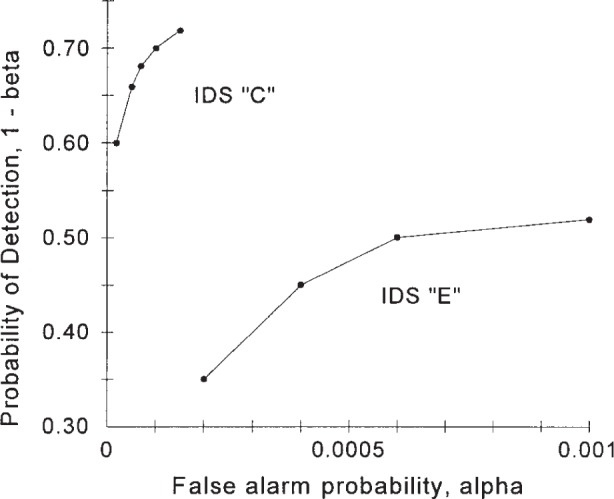
ROC curves.

Figure 1 shows two possible ROC curves that are used in this paper. These are similar to two ROC curves that were determined by Graf et al. [8] from actual data in the 1998 DARPA off-line intrusion detection evaluation. IDS E’s ROC curve is similar to the ROC curve for the EMERALD (Event Monitoring Enabling Responses to Anomalous Live Disturbances [9]), and IDS C’s ROC curve is similar to the ROC curve for the Columbia IDS [10]. IDS “C” is shown with five discrete operating points, and IDS “E” is shown with four. The lines shown connecting the points are added as a visual aid to the reader but are irrelevant to describing the performance of the IDSs. Gaffney and Ulvila [11] show that one would never choose to operate an IDS at an interior point on the line segment connecting two operating points.

The following nomenclature is used throughout this paper. The system can be in one of two states or conditions: either with an intrusion present (I) or with no intrusion present (NI). The prior probability of an intrusion is called *p*. The IDS reports either an intrusion alarm (A) or no alarm (NA). The parameters of the IDS’s ROC curve are: the probability of an alarm given an intrusion, the detection probability, *P*(A|I) = 1 − *β* (or the probability of no alarm given an intrusion, *P*(NA|I) = *β*), and the probability of an alarm given no intrusion, the false alarm probability, *P*(A|NI) = *α*. Thus, *α* and *β* are the probabilities of the two types of reporting errors.

Either report from the IDS will trigger one of two actions: either respond as though there were an intrusion (R) or do not respond (NR). Consequences of the combinations of possible actions and states of the system are specified by the costs of errors. The cost of responding as though there were an intrusion when there is none is denoted *C_α_*. The cost of failing to respond to an intrusion is denoted *C_β_*. Without loss of generality, we can rescale costs by defining a cost ratio, *C* = *C_β_*/*C_α_*. The analyses in the body of this paper assume that the costs of correct responses are zero. Section 8.3 describes how these analyses could be extended to the general situation with costs for all combinations of actions and states of the system.

In practice, these costs are estimated by considering the consequences of the errors, and costs will be different for different computer systems and for different operating conditions. For example, *C_α_* includes the obvious cost of the person who responds to the alarm and the not-as-obvious cost to the users due to the degraded performance of the computer system while the alarm is being investigated. These costs depend on the nature of the response. Common responses include: filtering, isolation, changing logging or other procedures, or disconnection [1], and some of the responses could be automated. *C_β_* is the cost of the damage done by the intruder while he remains undetected. It includes the cost to restore the computer system to its undamaged condition. For critical systems, it could include the costs of errors committed by the system while under the influence of the intruder (e.g., launching a missile or shutting down a power grid). In the analysis presented here, point-estimates are used for costs. An extension could use probability distributions over the costs, but the results, which are based on expected costs, would be similar.

In general, companies are reluctant to share information about their costs, but a procedure such as the following could be used by an organization to estimate these costs. The cost of various actions, such as responding to an alarm might be estimated by a careful consideration of the steps that would be taken to respond to one. The cost of ignoring an alarm when there actually is an intrusion into the system might be estimated in part by an analysis of the data available from surveys such as the 2002 CSI/FBI Computer Crime and Security Survey [12], and in part by a careful analysis of the cost or impact on the system and organization protected by the IDS. Industry data, such as those available from a survey, can suggest a value or range of values. However, such “industry data” cannot be a completely satisfactory substitute for a careful analysis of one’s own organization or business. This situation is analogous to the estimation of software development costs. One might use “canned” data, such as available from a commercial tool or what one obtains from discussions with other organizations’ personnel or from published papers or books. However, it is always preferable to use data from one’s own organizational experience as the basis of an estimate.

**Fig. 2:**
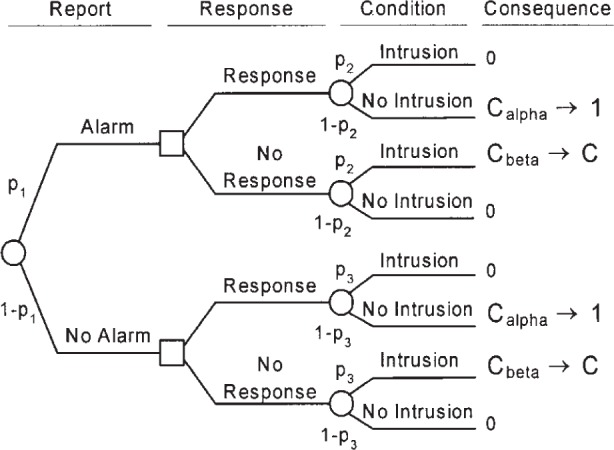
Decision tree of the IDS’s expected cost.

The expected cost of any operating point of the IDS is determined by analyzing the decision tree shown in Fig. 2. This decision tree shows the sequence of actions (squares) and uncertain events (circles) that describe the operation of the IDS and of the actions or responses that can be taken, based on reports. It also shows the consequences of the combinations of actions and events. The costs shown correspond to the consequences. The convention in a decision tree is to read it from left to right. The path leading to any point in the tree is shown to the left of the point and is assumed to be determined. Paths to the right of any point show all subsequent possibilities, which are not yet determined.

This decision tree shows that the optimal decision may be to take the action opposite of the one recommended by the IDS. That is, it may be optimal to ignore an alarm or to respond to a case of no alarm. Section 8.1 describes the conditions under which the optimal decision is to follow the IDS’s recommendation.

Decision or action nodes, which are displayed as squares, are under the control of the decision maker. The decision maker will choose which branch to follow. Event nodes, which are shown as circles, are not under the control of the decision maker but are subject to uncertainty. A probability distribution represents the uncertainty about which branch will happen following an event node. Associated with each uncertain event is its probability of occurrence. There are three probabilities specified in the tree:

*p*_1_ = the probability that the IDS reports an alarm,
*p*_2_ = the conditional probability of intrusion given that the IDS reports an alarm, and
*p*_3_ = the conditional probability of intrusion given that the IDS reports no alarm.

Gaffney and Ulvila [11] show how these probabilities can be derived from the values of *α*, β, and *p*.

The expected cost of an operating point is calculated by “rolling back” the decision tree [13] shown in Fig. 2. Working from right to left, the expected value at an event node is calculated as the sum of products of probabilities and costs for each branch. The expected cost at an action node is the minimum of expected costs on its branches.

An operating point for an IDS is defined as the values of the parameters *α* and *β*. Gaffney and Ulvila [11] show that the expected cost of operating at a point on an IDS’s ROC curve is: Min{*Cβ p*, (1 − *α*)(1 − *p*)} + Min{*C*(1 − *β*)*p*, *α*(1 − *p*)}, where *C* = *C_β_*/*C_α_* and *p* is the prior probability of intrusion.

Choosing the best operating point is important because IDSs can often be adjusted to operate at different points. Lippmann et al. [3] state: “most intrusion detection systems provide some degree of configuration to allow experts to customize the system to a given environment.” Axelsson [6] notes: “the performance point of the IDS can be tuned to meet the requirements of the operating environment.” Kent [14] states: “many systems have the equivalent of a tuning knob that allows a system administrator to adjust the sensitivity of the [intrusion detection system].”

The decision of choosing an operating point is to select the point with the least expected cost. That is, the values of *α* and *β* are chosen to minimize expected cost. The problem is to choose *α* and *β* on the ROC curve so as to minimize (for given values of *C* and *p*): Min{*Cβp*, (1 − *α*)(1 − *p*)} + Min{*C*(1 − *β*)*p*, *α*(1 − *p*)}.

**Fig. 3:**
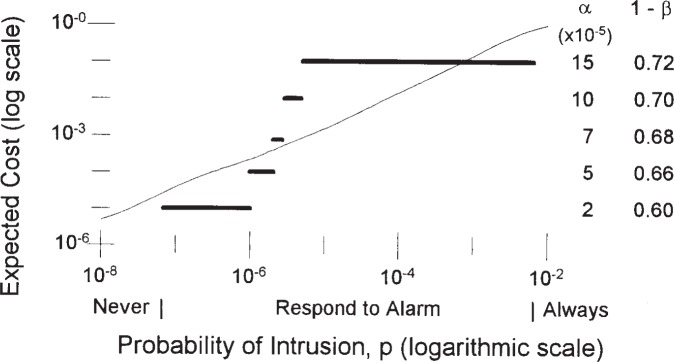
Optimal operating points and expected cost for IDS “*C*” (when cost ratio is 500).

Figure 3 shows, for IDS “*C*”, the relationship between the optimal operating point and the environment in which the IDS is to operate and the expected cost of operating at that point. It also shows the optimal response to an alarm. Figure 3 was determined for a cost ratio of 500. That is, if it is 500 times as expensive to fail to respond to an intrusion as it is to respond to a false alarm. Labels beneath the horizontal axis in Fig. 3 indicate that if the prior probability that a given attempt to use the system is an intrusion is less than 6.7 × 10^−8^, then it is best to never respond to an alarm. However, if the prior probability of an intrusion is greater than 7.1 × 10^−3^, then it is best to treat every attempt to use the system as though it were an intrusion. In between, it is best to respond to an alarm from the IDS.

The solid lines in Fig. 3 show the ranges over which the optimal operating point is the one shown on the right vertical axis. For example, if the prior probability of an intrusion is between 6.7 × 10^−8^ and 1.0 × 10^−6^, then the optimal operating point is *α* = 2 × 10^−5^ and 1 − *β* = 0.60. Continuing, if the prior probability of an intrusion is between 1.0 × 10^−6^ and 2.0 × 10^−6^, then the optimal operating point is at *α* = 5 × 10^−5^ and 1 − *β* = 0.66, and so forth.

The curve in Fig. 3 shows the expected cost (along the left vertical axis), in units of the cost of a false alarm, for each attempt to use the system when the IDS is operating at the optimal point and the optimal response decision is taken. The cost rises from 5.0 × 10^−6^ when the prior probability of intrusion is 1.0 × 10^−8^ to 1.4 × 10^−2^ when the prior probability of intrusion is 1.0 × 10^−4^ to 0.99 when the prior probability of intrusion is 1.0 × 10^−2^ (scales are logarithmic in Fig. 3).

The environment at the 1998 DARPA Off-line Intrusion Detection Evaluation was meant to simulate realistic normal traffic on a computer network at an Air Force base [1]. In this environment, there were 43 intrusion attempts out of 660 000 network sessions in a one-day period. This translates to a base-rate of intrusion of 43/660 000 = 6.52 × 10^−5^ per session. If the IDS is applied each session and intrusion responses are on a per-session basis, then, if we estimate the prior probability of intrusion as the base-rate, *p* = 6.52 × 10^−5^. Figure 3 shows that, at this prior probability of intrusion, the best decision is to respond to an alarm from the IDS, the expected cost is 0.009, and the best setting for the IDS is at *α* = 15 × 10^−5^ and 1 − *β* = 0.72.

The expected costs of different IDSs can be compared by subtracting the expected costs for the IDSs when each is operating at its optimal point. For any given cost ratio, *C*, and prior probability of intrusion, *p*, the optimal operating point will be different for IDSs with different ROC curves. Furthermore, the expected costs will differ for different ROC curves. The difference in expected cost provides an expected value metric for comparing the two IDSs.

In practice, one might be faced with the choice from among several different IDSs that offer different performances that can be characterized by different ROC curves. The analysis presented here provides a way to determine which ROC curve, and thus which IDS, is best. It also quantifies the preference in terms of a difference in expected cost. The choice of a preferred ROC curve and the degree of that preference depend on the operating environment as characterized by *p* and *C*.

**Fig. 4:**
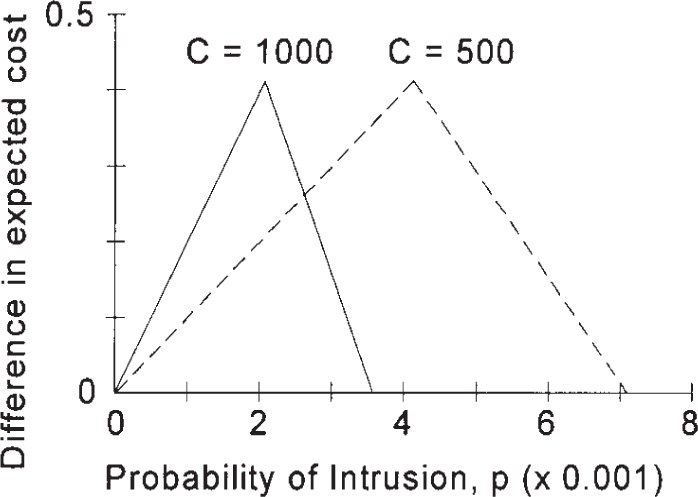
Expected value of IDS “*C*” over IDS “*E*” for different values of *C* and *p*.

Consider the two ROC curves shown in Fig. 1. Since the ROC curve for IDS “*C*” lies above and to the left of the ROC curve for IDS “*E*”, and since these curves do not intersect, IDS “*C*” is always better than IDS “*E*”. However, the value of that improvement, which is due to a smaller expected cost, depends on the values of *C* and *p*. Figure 4 summarizes the result. If *C* = 500 (i.e., if the cost of failing to respond to an intrusion is 500 times the cost of responding to a false alarm), then IDS “*C*” is preferred over IDS “*E*” for values of *p* less than 0.0071. The maximum difference in expected cost is 0.42 when *p* = .0042. If *C* = 1000, then IDS “*C*” is preferred for values of *p* less than 0.0036, and the maximum difference in expected cost is 0.42 when *p* = 0.0021.

## 3. Evaluation of Multiple Intrusion Detection Systems (IDSs)

**Fig. 5:**
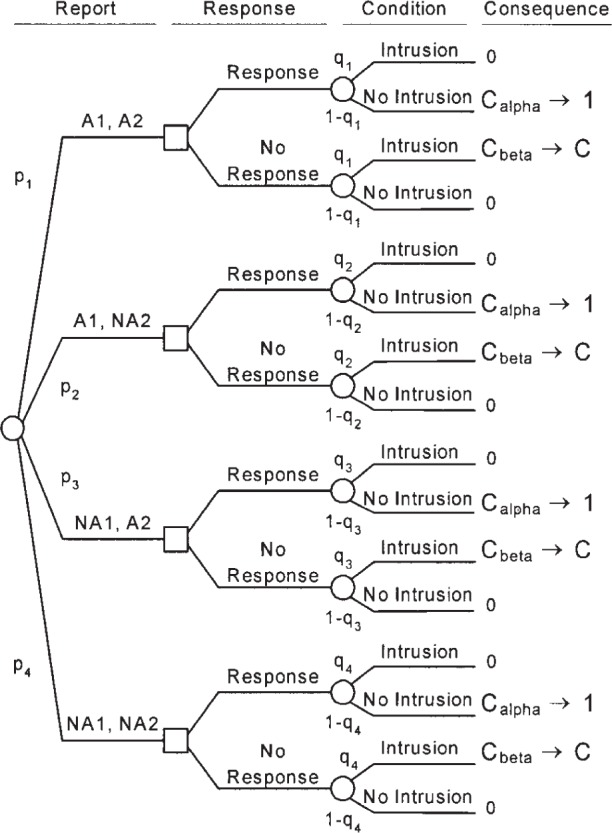
Decision tree for a compound IDS consisting of two IDSs.

The same type of analysis can be used to evaluate multiple IDSs operating in series or in parallel to evaluate the traffic on a system. In the case of two IDSs operating in a manner such that the results from both IDSs are known before the decision of whether to respond is made, the decision tree is as shown in Fig. 5. (Appendix B, Sec. 7, shows that the results are the same regardless of whether a single response decision is made on the basis of both IDSs’ reports or if response decisions are made sequentially after the receipt of each IDS’s report.) This decision tree is read the same way as the decision tree for a single IDS. The first uncertainty is the report from each IDS, an alarm or no alarm from IDS 1 (A1 or NA1) and IDS 2 (A2 or NA2). Next is the decision to respond or not. The next uncertainty is whether the actual condition is either an intrusion or no intrusion. Costs are the costs of errors—either responding to a false alarm (*C_α_*) or failing to respond to an intrusion (*C_β_*). The cost ratio, *C* = *C_β_*/*C_α_*. The parameters of this analysis are the probabilities of the reports, *p*_1_, *p*_2_, *p*_3_, and *p*_4_ and the probabilities of intrusion conditional on the reports, *q*_1_, *q*_2_, *q*_3_, and *q*_4_. Section 6 shows that, if the two IDSs are independent, then the expected cost for the two-IDS decision tree, in terms of the parameters of the two ROCs (*α*_1_, *α*_2_, *β*_1_, and *β*_2_), the prior probability of intrusion (*p*), and the cost ratio (*C*) is:

$$\mathrm{Min}\left\{\left(1–p\right)\alpha_{1}\alpha_{2},Cp\left(1–\beta_{1}\right)\left(1–\beta_{2}\right)\right\}+\mathrm{Min}\left\{\left(1–p\right)\alpha_{1}\left(1–\alpha_{2}\right),Cp\left(1–\beta_{1}\right)\beta_{2}\right\}+\mathrm{Min}\left\{\left(1–p\right)\left(1–\alpha_{1}\right)\alpha_{2},Cp\beta_{1}\left(1–\beta_{2}\right)\right\}+\mathrm{Min}\left\{\left(1–p\right)\left(1–\alpha_{1}\right)\left(1–\alpha_{2}\right),Cp\beta_{1}\beta_{2}\right\}.$$

### 3.1 Two Identical IDSs

**Fig. 6:**
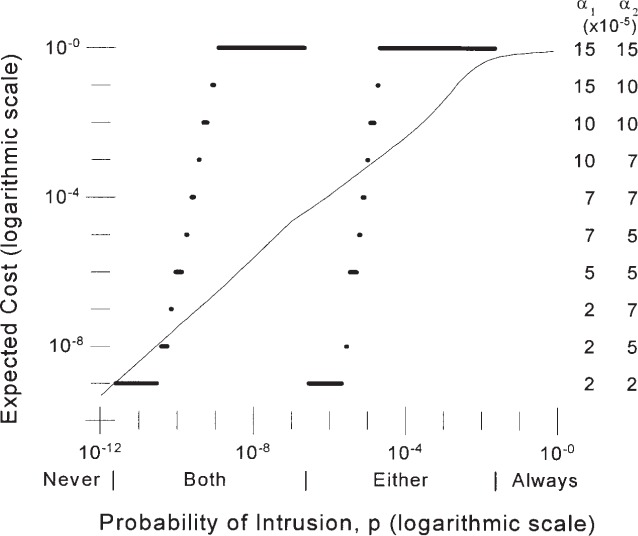
Results for two identical IDSs like IDS “*C*” (when cost ratio = 500).

The results of the analysis for two IDSs can be displayed in a fashion similar to the results for a single IDS. Figure 6 shows the results for two IDSs with identical, independent ROCs, when each IDS has the performance of IDS “*C*” and the cost ratio is 500. This could be the case if two IDSs used completely different methods of detection yet provided identical performance as evidenced by identical ROC curves. The stream of incoming traffic could be examined separately by each IDS, and each IDS would provide a separate alarm. Figure 6 shows the relationship between the optimal operating point and the environment in which the IDS is to operate and the expected cost of operating at that point. It has some interesting properties when compared with the analogous Fig. 3 for a single IDS. First, the “double IDS” is better than none over a larger range on the prior probability of intrusion, *p*, from 1.0 × 10^−11^ to 0.025. If *p* is below the lower limit, it is best to never respond to an alarm. If *p* is higher than the upper limit, it is best to respond to every attempt to use the system as though it were an intrusion. In between these limits, it is best to respond only if both IDSs indicate an alarm for values of *p* up to 2.5 × 10^−6^, and to respond to an alarm from either IDS above this value of *p*.

In the case of two IDSs, each IDS can be set independently so that the combined performance is optimal. This results in two different settings, one for each IDS. As the prior probability of intrusion increases, the optimal settings of the false alarm probabilities of the two IDSs (*α*_1_ and *α*_2_) increase as shown by the right-hand axis in Fig. 6. (See Fig. 1 for the value of 1 − *β* at each value of *α*.) Increases are usually changes in a single IDS’s setting, but sometimes the settings for both IDSs change. Once the value of *p* increases above 2.5 × 10^−7^, the optimal false alarm rates revert to their minima and begin to rise again as *p* continues to rise.

The curve in Fig. 6 shows that the expected cost (the left axis), in units of the cost of a false alarm, for each attempt to use the system when the two IDSs are operating rises as *p* rises.

Consider again the environment of the 1998 DARPA Off-line Detection Evaluation to estimate the value of *p* = 6.52 × 10^−5^. Figure 6 shows that, at this prior probability of intrusion, the best decision is to respond to an alarm from either IDS, the expected cost is less than 0.003, which is less than a third the expected cost on a single IDS, and the best setting for each IDS is at *α* = 15 × 10^−5^ (with 1 − *β* = 0.72).

**Fig. 7:**
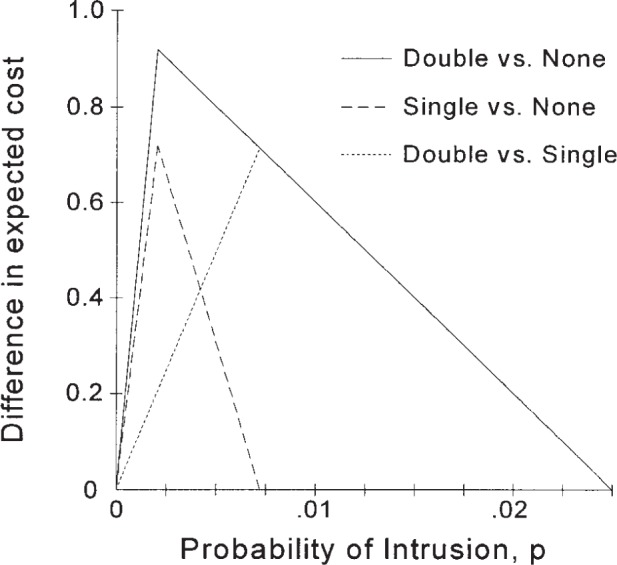
Expected value of two identical IDSs over one and none for different values of *p* (at *C* = 500).

The results from the analysis with two IDSs can be compared with the results for a single IDS as shown in Fig. 7. As can be easily seen, two IDSs are better than one over the whole range that two are better than none. The maximum difference in the value of two over one occurs at the point where the single IDS is no better than no IDS, i.e., at *p* = 0.007.

This result shows the limitations of the “convex hull” approach to evaluating multiple IDSs. Provost and Fawcett [15] recommend evaluating multiple IDSs by finding the convex hull of their ROC curves. They then argue that this convex hull represents the performance that could be gained from using both IDSs. If any part of an IDS’s ROC curve is on the convex hull of all ROC curves, then that IDS is the best one to use for some combination of *p* and *C*, the prior probability of intrusion and the cost ratio. However, their method fails to account for the synergistic effect that multiple IDSs offer to provide a more effective ROC curve than any single curve. Furthermore, only crossing ROC curves will produce different parts of the convex hull from different IDSs. Identical IDSs do not cross, so the convex hull is the same as the single IDS. Yet two IDSs are clearly better than one. The following section illustrates this more dramatically, when a dominated IDS is added and the two are better than either one individually.

### 3.2 Two Different IDSs

A similar analysis could be conducted for two different, independent IDSs. This is the more likely case, since it is more likely to find two independent IDSs with different ROC curves than with identical ROC curves. Suppose, for instance, that both IDS “*C*” and IDS “*E*” from Fig. 1 were available for use and that each provided an independent assessment of whether an attempt to use the system was an intrusion or not. We saw in Sec. 2 that IDS *C*’s performance dominated that of IDS “*E*”. However an analysis of the double IDS with both shows that both can be used to provide a lower expected cost than either.

**Fig. 8:**
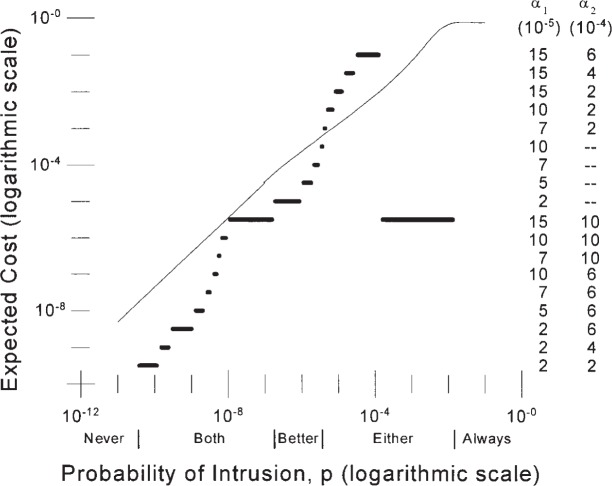
Results for two different IDSs like IDSs *C* and *E* (when cost ratio = 500).

The optimal operating points and expected cost of the double IDS with both IDS “*C*” and IDS “*E*” are shown in Fig. 8. With *C* = 500, the combination of the two different IDSs is better than none over a range on the prior probability of intrusion, *p*, from 3.8 × 10^−11^ to 0.015. If *p* is below the lower limit, it is best to never respond to an alarm. If *p* is higher than the upper limit, it is best to respond to every attempt to use the system as if it were an intrusion. In between these limits, the IDS with two different IDSs behaves slightly differently from the one with identical IDSs. For values of *p* up to 1.75 × 10^−7^ it is best to respond only if both IDSs give an alarm; as *p* increases above this value up to 3.7 × 10^−6^ it is best to respond only if IDS “*C*” (the better IDS) gives an alarm; above this value of *p*, it is best to respond to an alarm from either IDS.

As the prior probability of intrusion increases, the optimal settings of the false alarm probabilities of the two IDSs change as shown by the right-hand axis in Fig. 8. As the value of *p* increases above 1.75 × 10^−7^, the optimal false alarm rate of the better IDS (IDS “*C*”) reverts to its minimum. As the value of *p* continues to increase above 3.7 × 10^−6^, the optimal false alarm rates for both IDSs drop and begin to rise again as *p* continues to rise.

Consider again the environment of the 1998 DARPA Off-line Detection Evaluation to estimate the value of *p* = 6.52 × 10^−5^. Figure 8 shows that, at this prior probability of intrusion, the best decision is to respond to an alarm from either IDS, and the expected cost is about 0.005, which is a little over half the expected cost with a single IDS like IDS “*C*”. At this prior probability of intrusion, the best setting for IDS “*C*” is at *α* = 15 × 10^−5^ (with 1 − *β* = 0.72) and for IDS “*E*” is at *α* = 6 × 10^−4^ (with 1 − *β* = 0.50).

**Fig. 9:**
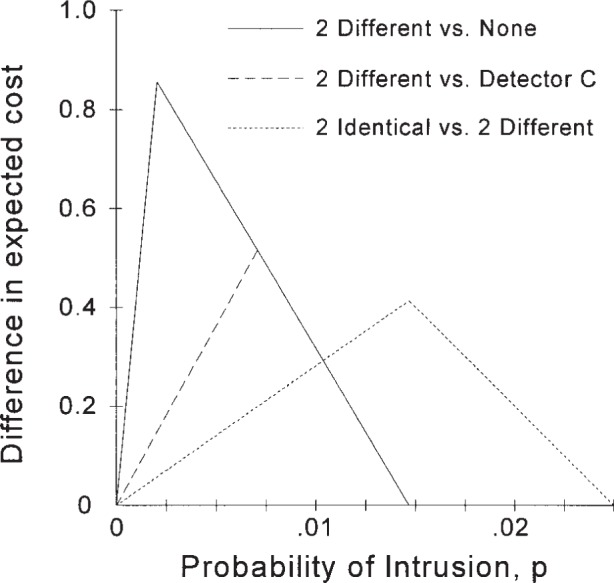
Analysis of two different IDSs (at *C* = 500).

The results from the analysis with two different IDSs can be compared with the results for the single better IDS (IDS “*C*”) as shown in Fig. 9. As can be easily seen, even though IDS “*E*” is dominated by IDS “*C*”, the two different IDSs used together are better than IDS “*C*” over the whole range that the two are better than none (except for the range where the optimal decision is to respond only to alarms from IDS “*C*,” in which case the double IDS has the same expected cost as IDS “*C*”). The maximum difference in the value of two different IDSs over IDS “*C*” occurs at the point where IDS “*C*” is no better than no IDS, i.e., at *p* = 0.007. Figure 9 also shows that the double IDS made up of two IDSs identical to IDS “*C*” (the better part of the duo) is better than the double IDS made up of the two different IDSs “*C*” and “*E*”.

### 3.3 Two IDSs, One With No False Alarms

Wagner and Dean [16] describe an approach to intrusion detection using static analysis. They claim three advantages: a high degree of automation, protection against a broad class of attacks, and elimination of false alarms. The most important class of attacks for which their approach is applicable is buffer overflows, which accounted for at least half of 1999 CERT advisories [17]. They also claim that their approach is able to detect Trojan horses in trusted software, any dynamic-linking attack, and format string attacks. They recommend that their approach “should not be used as the sole defense against any of these attacks, but instead should be used to complement other techniques.” This makes it an ideal case to study in conjunction with one of the IDSs describe in this paper.

Wagner and Dean [16] do not give any detection probability information in their paper. In a subsequent e-mail to one of the authors, Wagner stated that no estimate of detection probability is available. So, for purposes of this analysis, assume that an IDS based on static analysis could detect 60 % of the attack attempts with a conditional probability of detection of 0.50. Thus, when considering all attack possibilities, an IDS based on this approach would have an operating point of *α* = 0.00 at 1 − *β* = (0.60)(0.50) = 0.30.

**Fig. 10:**
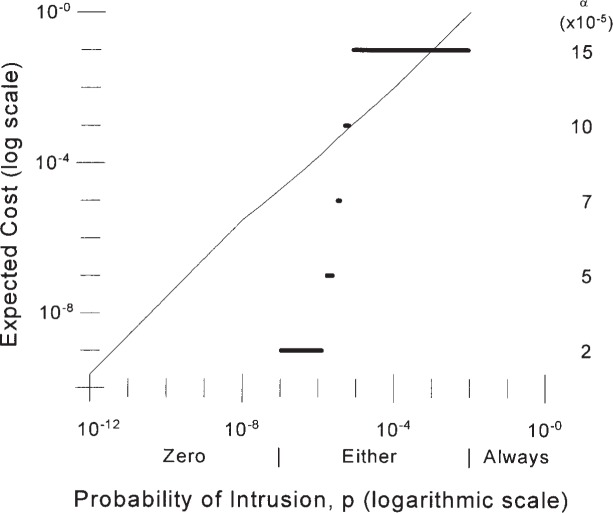
Results for two IDSs, one with zero false alarms (when cost ratio = 500).

Consider the operation of an intrusion detection system that consists of a Wagner and Dean [16] “zero false alarm” IDS combined with IDS “*C*” from Fig. 1. The optimal operating points and expected cost from optimal operation of such a system are shown in Fig. 10. With *C* = 500, the combination of IDS “*C*” with the “zero false alarm” IDS is better than no IDS over a range on the prior probability of intrusion, *p*, from 0.00 to 0.010. If *p* is higher than the upper limit, it is best to respond to every attempt to use the system as if it were an intrusion. However, because one of the component IDSs offers a positive detection probability at a zero false alarm probability, it is always better to respond to an alarm from the “zero false alarm” part of the system than to ignore it. For values of *p* up to 9.5 × 10^−8^, it is best to respond only if the “zero false alarm” IDS gives an alarm. For values of *p* between 9.5 × 10^−8^ and 0.010, it is best to respond if either IDS gives an alarm. For values of *p* above 0.010, it is best to respond to every attempt to use the system as though it were an intrusion.

The “zero false alarm” IDS should always be operated at its ROC point (0.00, 0.30). As the prior probability of intrusion increases, the optimal settings of the false alarm probabilities of the IDS “*C*” portion of the IDS changes as shown by the right-hand axis in Fig. 10.

Consider again the environment of the 1998 DARPA Off-line Detection Evaluation to estimate the value of *p* = 6.52 × 10^−5^. Figure 10 shows that, at this prior probability of intrusion, the best decision is to respond to an alarm from either IDS, and the expected cost is about 0.0065, which is about two-thirds the expected cost with a single IDS “*C*”. At this prior probability of intrusion, the best setting for the IDS “*C*” portion of the system is at *α* = 15 × 10^−5^ (with 1 - *β* = 0.72).

**Fig. 11:**
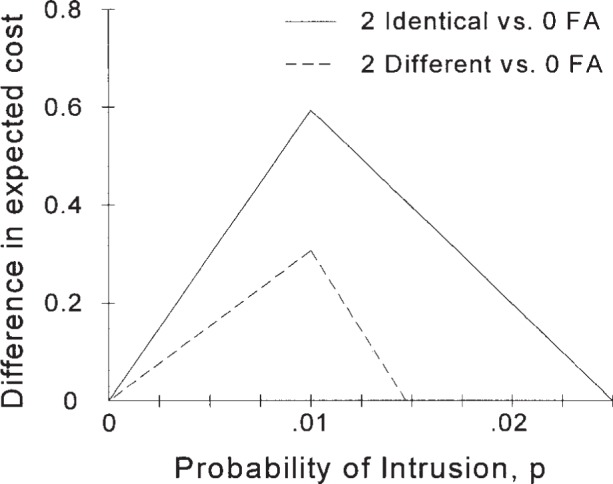
Comparisons of dual IDSs (at *C* = 500).

The results from the analyses of all three dual IDSs are shown in Fig. 11. For very low prior probabilities of intrusion, below about 2 × 10^-5^ for the dual IDS with IDSs “*C*” and “*E*” and 1 × 10^-11^ for dual identical IDS “*C*”s, the dual IDS with a “zero false alarm” (0 FA) IDS is better than one or both of the other dual IDSs analyzed in this paper. Both of the others are better (as long as they are better than no IDS) for probabilities above 2 × 10^-5^. The maximum difference in expected value (if *C* = 500) is at *p* = 0.01, the point at which the dual IDS with a “zero false alarm” component is no better than no IDS.

## 4. Composite ROC Curve For Multiple IDSs

Results from the analyses in Sec. 3 indicate that it might be possible to represent the performance of two independent IDSs in a single, composite ROC curve. That is, since the expected cost and ranges on optimal operating points from the analysis of two independent IDSs can be represented in the same manner as those results for a single IDS, it might be possible to summarize the performance of the two IDSs in one ROC curve. Section 9 shows that it is indeed possible to derive a single, composite ROC curve from the ROC curves of two independent IDSs.

The composite ROC curve displays the performance of the combination of two component IDSs in two parameters, *α* and *β*. This composite ROC curve is interpreted in the same way as any ROC curve. The values of *α* and *β* are functions of the parameters of the component IDSs, *α*_1_, *β*_1_, *α*_2_, and *β*_2_. The functional form, however, depends on the optimal decision rule used to respond to alarms from the component IDSs. Recall that the decision rule could be any one of the following: 1) respond only if both component IDSs indicate an alarm, 2) respond only if one particular component IDS indicates an alarm, 3) respond only if the other component IDS indicates an alarm, or 4) respond if either IDS indicates an alarm.

In cases where the optimal decision rule is to respond to a single IDS’s alarm, the values of *α* and *β* are equal to those of the single IDS whose advice is followed. When the optimal decision rule is to respond only when both component IDSs indicate an alarm, the parameters of the composite ROC curve are: *α* = *α*_1_*α*_2_, and *β* = *β*_1_ + *β*_2_ − *β*_1_*β*_2_. When the optimal decision rule is to respond to an alarm from either component IDS, the effective parameters of the composite ROC curve are: *α* = *α*_1_ + *α*_2_ − *α*_1_*α*_2_, and β = *β*_1_*β*_2_. In any case, the expected cost (EC), in terms of the prior probability of intrusion, *p*, and the cost ratio, *C*, from operating at a point on the ROC curve is: *EC* = (1 − *p*)*α* + *Cpβ*.

**Fig. 12:**
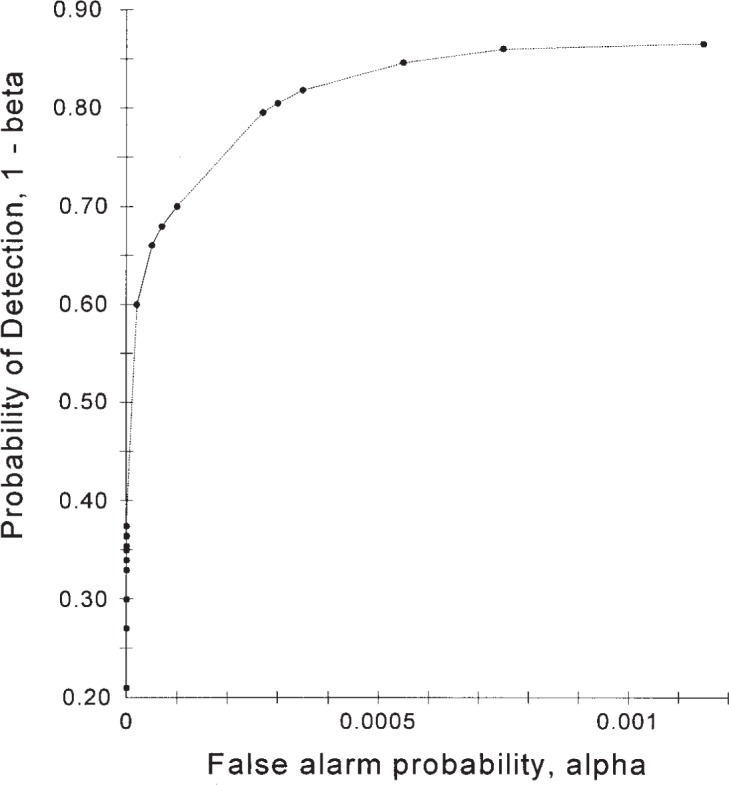
Composite ROC curve for an IDS consisting of IDS “*C*” and IDS “*E*”.

Applying these results to the analysis with two different IDSs from Sec. 3.2 gives the composite ROC curve with the points shown in Table 1. This ROC curve is displayed graphically in Fig. 12.

Notice that four points from the ROC of IDS “*C*,” which are shown bounding the solid lines in Fig. 12, are points on the composite ROC. The fifth point (15 × 10^−5^, 0.72) is not on the composite ROC because it is not on the convex hull of all points. Section 8.2 describes how the convex hull of points is determined from a set of points. All possible points for the composite ROC are generated by considering all four decision rules.

Since the performance of a compound IDS composed of two independent IDSs can be represented as a single composite ROC curve, its analysis can be performed in exactly the same way as the analysis of a single IDS. That is, an analysis of the ROC curve in Fig. 12 produces the same results as displayed in Fig. 8 (in Sec. 3.2). Furthermore, the composite ROC curve for two IDSs can be combined with the ROC curve from another independent IDS to produce a composite ROC curve for the combined IDSs. That is, the same method used to combine two independent ROC curves can be applied iteratively to combine any number of independent ROC curves.

This finding suggests the following, six-step procedure to evaluate any number of independent IDSs:

Step 1. Determine the equivalent convex ROC curve for each IDS. (See Sec. 8.1 for the method to determine the convex equivalent for concave sections of an ROC curve.)
Step 2. For a pair of IDSs, specify all combinations of points on the two ROC curves.
Step 3. Determine the values of parameters *α* and *β* for all combinations of points from Step 2 under each of these two decision rules: 1) respond only if both IDSs indicate an alarm and 2) respond if either IDS indicates an alarm.
Step 4. Identify the convex hull of the set of all points. The set consists of the points (*α*, 1 − *β*) on each component IDS’s ROC curve, all of the points determined in Step 3, and the endpoints, (0, 0) and (1, 1). The convex hull of these points is the composite ROC curve for the two independent IDSs.
Step 5. Repeat Steps 2 through 4 until all component IDSs are included in the composite ROC curve. This might be done by adding each component’s ROC curve, in turn, to the composite ROC curve or by first combining pairs of ROC curves and then combining the resulting composite curves.
Step 6. Use the final composite ROC curve to analyze the combined performance of all of the independent IDSs. Notice that one must keep track of the combination rules used to generate each point on the composite ROC curve in order to determine the settings of the component IDSs that produce each point.

## 5. Conclusions, Recommendations, and Extensions

The analysis in this paper demonstrates that the most commonly recommended methods for evaluating and comparing IDSs are flawed. IDSs should not be evaluated based on the areas under their ROC curves, their distances from a goal, or their false alarm rates. Evaluations should be based on expected costs that reflect: the cost of a false alarm, the cost of a failure to detect (or a ratio of these costs), and the prior probability of intrusion. Furthermore, the operating point of an IDS, its probability of a false alarm (*α*) and probability of missed detection (*β*), should be established to minimize the expected cost.

When considering the operation of multiple IDSs, the ROC convex hull [15] is an insufficient guide for determining how to make best use of multiple IDSs. The ROC convex hull can be used to determine the combinations of costs and prior probabilities of intrusion for which one IDS is preferred over another. It does not indicate the degree of preference. However, unless one of the IDSs is worthless, it is better to use the IDSs in combination than to use a single IDS. The performance of the combination of IDSs can be represented in a composite ROC curve that can be used for analyses.

The methods described in this paper are suitable for development into a decision support tool that could be used by a system administrator to choose among IDSs, to indicate the best use of a single IDS or any combination of independent IDSs, and to set the operating parameters of an IDS for optimal performance in a given environment characterized by costs and the prior probability of intrusion. This tool could also be used by IDS developers to evaluate design tradeoffs that lead to different performance.

Different types of intrusions can be analyzed by extending the decision tree in Fig. 2 to include event nodes that show explicitly the type of intrusion with a different ROC curve applied to each. For example, ROC curves for the Columbia IDS were determined for the four types of attacks included in the 1998 DARPA off-line intrusion detection evaluation [3,4,10]. Columbia’s ROC curve is worst against denial of service attacks, a little better against remote-to-local and user-to-root attacks (the same ROC curve applies for both of these types of attacks), and substantially better against surveillance and probing attacks.

The analysis in this paper handles an evolving attack of intrusion and response only implicitly. The probability of detection is interpreted as the probability that an intrusion is detected before it does any damage, and the response is interpreted to be both immediate and effective. Any intrusion that is not detected and countered immediately and effectively is modeled in the single path with no response to an intrusion. That is, *C_β_* is the expected cost considering all levels of damage that may occur before the intrusion is neutralized. A more detailed analysis could show a time sequence of events that corresponded to delayed detections, delayed responses, their probabilities, and their effectiveness.

Although the analysis in this paper concentrates on the case where costs are associated only with errors, the method extends to analyses with other costs, as outlined in Sec. 8.

## Figures and Tables

**Fig. 13:**
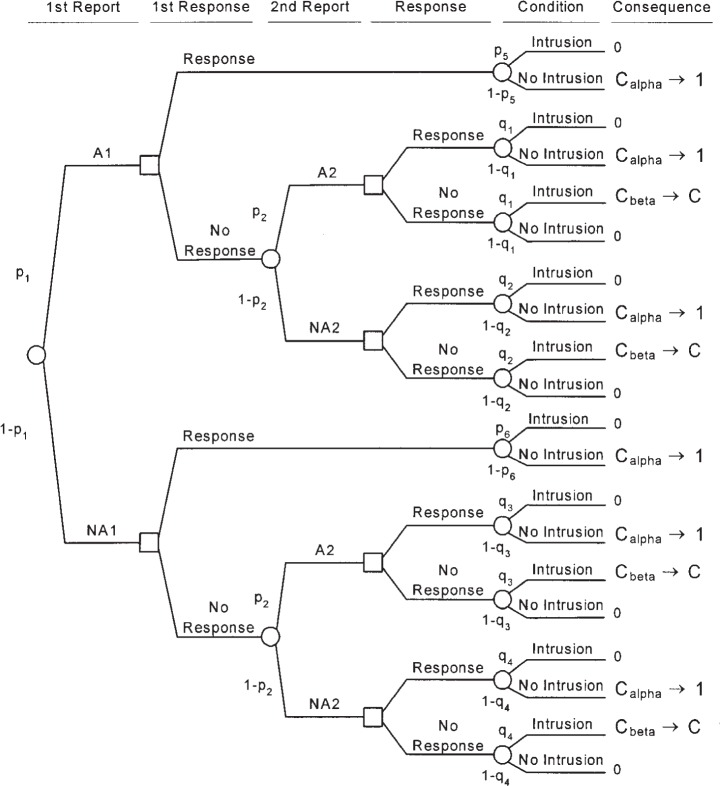
Two detectors, sequential decisions.

**Fig. 14:**
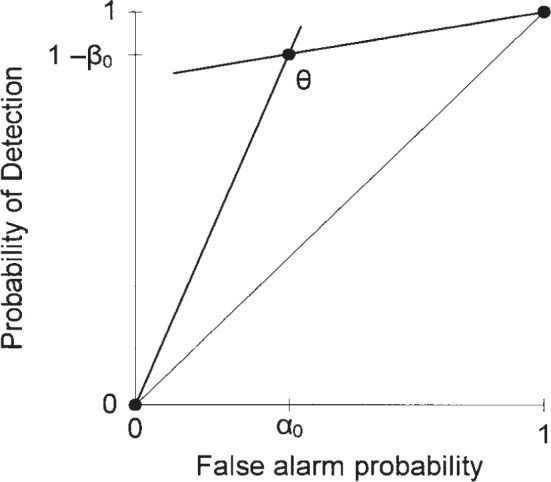
Convex hull of a ROC curve with a single point.

**Fig. 15:**
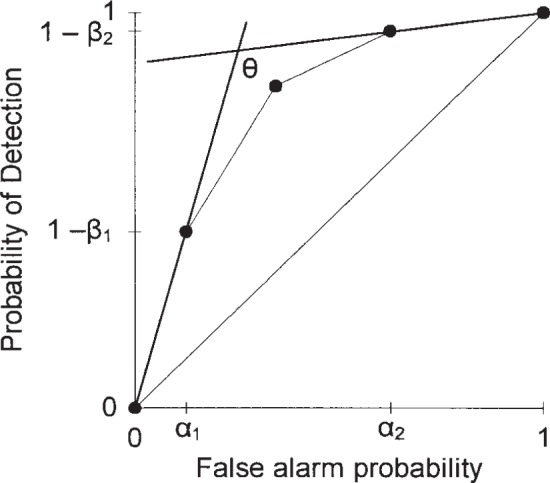
Convex hull of a ROC curve with multiple points.

**Fig. 16:**
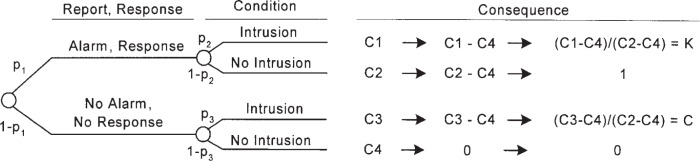
Event tree with general costs.

**Table 1:** Composite ROC curve for two different IDSs

IDS 1 (IDS “*C*”)		IDS 2 (IDS “*E*”)			Composite	
*α*_1_	1−*β*_1_	*α*_2_	1−*β*_2_	Respond to:	*α*	1−*β*
1.00	1.00	1.00	1.00	Always	1.00	1.00
15 × 10^−5^	0.72	10 × 10^−4^	0.52	Either	115.0 × 10^−5^	0.8656
15 × 10^−5^	0.72	6 × 10^−4^	0.50	Either	75.0 × 10^−5^	0.8600
15 × 10^−5^	0.72	4 × 10^−4^	0.45	Either	55.0 × 10^−5^	0.8460
15 × 10^−5^	0.72	2 × 10^−4^	0.35	Either	35.0 × 10^−5^	0.8180
10 × 10^−5^	0.70	2 × 10^−4^	0.35	Either	30.0 × 10^−5^	0.8050
7 × 10^−5^	0.68	2 × 10^−4^	0.35	Either	27.0 × 10^−5^	0.7920
10 × 10^−5^	0.70			IDS 1	10.0 × 10^−5^	0.7000
7 × 10^−5^	0.68			IDS 1	7.0 × 10^−5^	0.6800
5 × 10^−5^	0.66			IDS 1	5.0 × 10^−5^	0.6600
2 × 10^−5^	0.60			IDS 1	2.0 × 10^−5^	0.6000
15 × 10^−5^	0.72	10 × 10^−4^	0.52	Both	15.0 × 10^−8^	0.3744
10 × 10^−5^	0.70	10 × 10^−4^	0.52	Both	10.0 × 10^−8^	0.3640
7 × 10^−5^	0.68	10 × 10^−4^	0.52	Both	7.0 × 10^−8^	0.3536
10 × 10^−5^	0.70	6 × 10^−4^	0.50	Both	6.0 × 10^−8^	0.3500
7 × 10^−5^	0.68	6 × 10^−4^	0.50	Both	4.2 × 10^−8^	0.3400
5 × 10^−5^	0.66	6 × 10^−4^	0.50	Both	3.0 × 10^−8^	0.3300
2 × 10^−5^	0.60	6 × 10^−4^	0.50	Both	1.2 × 10^−8^	0.3000
2 × 10^−5^	0.60	4 × 10^−4^	0.45	Both	0.8 × 10^−8^	0.2700
2 × 10^−5^	0.60	2 × 10^−4^	0.35	Both	0.4 × 10^−8^	0.2100
0.00	0.00	0.00	0.00	Never	0.0	0.0000

## 6. Appendix A: Analysis For a Compound IDS With a Single Decision

This appendix shows the analysis for a compound IDS composed of two independent IDSs when the response decision is made only once on the basis of both reports. For two independent detectors:

$$\begin{array}{l} P\left(A1,A2|I\right)=P\left(A1|I\right)P\left(A2|I\right)=\left(1–\beta_{1}\right)\left(1–\beta_{2}\right)/p_{1};P\left(A1,A2|\mathrm{NI}\right)=P\left(A1|\mathrm{NI}\right)P\left(A2|\mathrm{NI}\right)=\alpha_{1}\alpha_{2};\\ P\left(A1,\mathrm{NA}2|I\right)=P\left(A1|I\right)P\left(\mathrm{NA}2|I\right)=\left(1–\beta_{1}\right)\beta_{2};P\left(A1,\mathrm{NA}2|\mathrm{NI}\right)=P\left(A1|\mathrm{NI}\right)P\left(\mathrm{NA}2|\mathrm{NI}\right)=\alpha_{1}\left(1–\alpha_{2}\right);\\ P\left(\mathrm{NA}1,A2|I\right)=P\left(\mathrm{NA}1|I\right)P\left(A2|I\right)=\beta_{1}\left(1–\beta_{2}\right);P\left(\mathrm{NA}1,\;A2|\mathrm{NI}\right)=P\left(\mathrm{NA}1|\mathrm{NI}\right)P\left(A2|\mathrm{NI}\right)=\left(1–\alpha_{1}\right)\alpha_{2};\\ P\left(\mathrm{NA}1,\mathrm{NA}2|I\right)=P\left(\mathrm{NA}1|I\right)P\left(\mathrm{NA}2|I\right)=\beta_{1}\beta_{2};P\left(\mathrm{NA}1,\;\mathrm{NA}2|\mathrm{NI}\right)=P\left(\mathrm{NA}1|\mathrm{NI}\right)P\left(\mathrm{NA}2|\mathrm{NI}\right)=\left(1–\alpha_{1}\right)\;\left(1–\alpha_{2}\right). \end{array}$$

So, by Bayes’ Theorem (see [13]), the values of the parameters are:

$$\begin{array}{l} q_{1}=P\left(A1,A2|I\right)P\left(I\right)/p_{1}=p\left(1–\beta_{1}\right)\left(1–\beta_{2}\right)/p_{1};\;1–q_{1}=P\left(A1,A2|\mathrm{NI}\right)P\left(\mathrm{NI}\right)/p_{1}=\left(1–p\right)\alpha_{1}\alpha_{2}/p_{1};\\ q_{2}=P\left(A1,\mathrm{NA}2|I\right)P\left(I\right)/p_{2}=p\left(1–\beta_{1}\right)\beta_{2}/p_{2};\;1–q_{2}=P\left(A1,\mathrm{NA}2|\mathrm{NI}\right)P\left(\mathrm{NI}\right)/p_{2}=\left(1–p\right)\alpha_{1}\left(1–\alpha_{2}\right)/p_{2};\\ q_{3}=P\left(\mathrm{NA}1,A2|I\right)P\left(I\right)/p_{3}=p\beta_{1}\left(1–\beta_{2}\right)/p_{3};\;1–q_{3}=P\left(\mathrm{NA}1,A2|\mathrm{NI}\right)P\left(\mathrm{NI}\right)/p_{3}=\left(1–p\right)\left(1–\alpha_{1}\right)\alpha_{2}/p_{3};\\ q_{4}=P\left(\mathrm{NA}1,\mathrm{NA}2|I\right)P\left(I\right)/p_{4}=p\beta_{1}\beta_{2}/p_{4};\;1–q_{4}=P\left(\mathrm{NA}1,\mathrm{NA}2|\mathrm{NI}\right)P\left(\mathrm{NI}\right)/p_{4}=\left(1–p\right)\left(1–\alpha_{1}\right)\left(1–\alpha_{2}\right). \end{array}$$

$$\text{Expected Cost}=p_{1}\mathrm{Min}\left\{1–q_{1},Cq_{1}\right\}+p_{2}\mathrm{Min}\left\{1–q_{2},Cq_{2}\right\}+p_{3}\mathrm{Min}\left\{1–q_{3},Cq_{3}\right\}+p_{4}\mathrm{Min}\left\{1–q_{4},Cq_{4}\right\}$$

$$\begin{array}{l} =p_{1}\mathrm{Min}\left\{\left(1–p\right)\alpha_{1}\alpha_{2}/p_{1},Cp\left(1–\beta_{1}\right)\left(1–\beta_{2}\right)/p_{1}\right\}+p_{2}\mathrm{Min}\left\{\left(1–p\right)\alpha_{1}\left(1–\alpha_{2}\right)/p_{2},Cp\left(1–\beta_{1}\right)\beta_{2}/p_{2}\right\}\;\\ +p_{3}\mathrm{Min}\left\{\left(1–p\right)\left(1–\alpha_{1}\right)\alpha_{2}/p_{3},Cp\beta_{1}\left(1–\beta_{2}\right)/p_{3}\right\}+p_{4}\mathrm{Min}\left\{\left(1–p\right)\left(1–\alpha_{1}\right)\left(1–\alpha_{2}\right)/p_{4},Cp\beta_{1}\beta_{2}/p_{4}\right\} \end{array}$$

$$\begin{array}{l} =\mathrm{Min}\left\{\left(1–p\right)\alpha_{1}\alpha_{2},Cp\left(1–\beta_{1}\right)\left(1–\beta_{2}\right)\right\}+\mathrm{Min}\left\{\left(1–p\right)\alpha_{1}\left(1–\alpha_{2}\right),Cp\left(1–\beta_{1}\right)\beta_{2}\right\}\\ +\mathrm{Min}\left\{\left(1–p\right)\left(1–\alpha_{1}\right)\alpha_{2},Cp\beta_{1}\left(1–\beta_{2}\right)\right\}+\mathrm{Min}\left\{\left(1–p\right)\left(1–\alpha_{1}\right)\left(1–\alpha_{2}\right),Cp\beta_{1}\beta_{2}\right\}. \end{array}$$

## 7. Appendix B: Analysis For a Compound IDS With Sequential Decisions

This appendix shows the analysis for a compound IDS composed of two independent IDSs when the response decision is made sequentially after each component IDS’s report. The decision tree for two IDSs with sequential decisions, one after each IDS’s report, is shown in Fig. 13.

The analysis is as follows. For two independent detectors:

$$\begin{array}{l} p_{1}=P\left(A1|I\right)P\left(I\right)+P\left(A1|\mathrm{NI}\right)P\left(\mathrm{NI}\right)=\left(1–\beta_{1}\right)p+\alpha_{1}\left(1–p\right);\;1–p_{1}=\beta_{1}p+\left(1–\alpha_{1}\right)\left(1–p\right);\\ p_{2}=P\left(A2|I\right)P\left(I\right)+P\left(A2|\mathrm{NI}\right)P\left(\mathrm{NI}\right)=\left(1–\beta_{2}\right)p+\alpha_{2}\left(1–p\right);\;1–p_{2}=\beta_{2}p+\left(1–\alpha_{2}\right)\left(1–p\right);\\ p_{5}=P\left(I|A1\right)=P\left(A1|I\right)P\left(I\right)/P\left(A_{1}\right)=\left(1–\beta_{1}\right)p/p_{1};\;1-p_{5}=\alpha_{1}\left(1-p\right)/p_{1};\\ p_{6}=P\left(I|\mathrm{NA}1\right)=P\left(\mathrm{NA}1|I\right)P\left(I\right)/P\left(\mathrm{NA}1\right)=\beta_{1}p/\left(1–p_{1}\right);\;1–p_{6}=\left(1–\alpha_{1}\right)\left(1–p\right)/\left(1–p_{1}\right). \end{array}$$

$$\begin{array}{l} q_{1}=P\left(I|A1,\;A2\right)=P\left(A1,A2|I\right)P\left(I\right)/P\left(A1,A2\right)=p\left(1–\beta_{1}\right)\left(1–\beta_{2}\right)/p_{1}p_{2};\;1–q_{1}=\left(1–p\right)\alpha_{1}\alpha_{2}/p_{1}p_{2};\\ q_{2}=P\left(I|A1,\;\mathrm{NA}2\right)=P\left(A1,\mathrm{NA}2|I\right)P\left(I\right)/P\left(A1,\mathrm{NA}2\right)=p\left(1–\beta_{1}\right)\beta_{2}/p_{1}\left(1–p_{2}\right);\\ \;1–q_{2}=\left(1–p\right)\alpha_{1}\left(1–\alpha_{2}\right)/p_{1}\left(1–p_{2}\right);\\ q_{3}=P\left(I|\mathrm{NA}1,\;A2\right)=p\beta_{1}\left(1–\beta_{2}\right)/\left(1–p_{1}\right)p_{2};\;1–q_{3}=\left(1–p\right)\left(1–\alpha_{1}\right)\alpha_{2}/\left(1–p_{1}\right)p_{2};\\ q_{4}=p\beta_{1}\beta_{2}/\left(1–p_{1}\right)\left(1–p_{2}\right);\;1–q_{4}=\left(1–p\right)\left(1–\alpha_{1}\right)\left(1–\alpha_{2}\right)/\left(1–p_{1}\right)\left(1–p_{2}\right). \end{array}$$

$$\begin{array}{l} \text{Expected}\;\text{Cost}=p_{1}\mathrm{Min}[\left(1–p\right)\alpha_{1}/p_{1},\;\mathrm{Min}\left\{\left(1–p\right)\alpha_{1}\alpha_{2}/p_{1}p,Cp\left(1–\beta_{1}\right)\left(1–\beta_{2}\right)/p_{1}p_{2}\right\}+\left(1–p_{2}\right)\\ \mathrm{Min}\left\{\left(1–p\right)\alpha_{1}\left(1–\alpha_{2}\right)/p_{1}\left(1-p_{2}\right),\;Cp\left(1–\beta_{1}\right)\beta_{2}/p_{1}\left(1\;–p_{2}\right)\right\}]+\left(1–p_{1}\right)\mathrm{Min}[\left(1–p\right)\left(1–\alpha_{1}\right)/\left(1–p_{1}\right),\\ \mathrm{Min}\left\{\left(1–p\right)\left(1–\alpha_{1}\right)\alpha_{2}/\left(1–p_{1}\right),Cp\beta_{1}\left(1–\beta_{2}\right)/\left(1-p_{1}\right)\right\}+\mathrm{Min}\left\{\left(1–p\right)\left(1–\alpha_{1}\right)\left(1–\alpha_{2}\right)/\left(1–p_{1}\right),Cp\beta_{1}\beta_{2}/\left(1–p_{1}\right)\right\}] \end{array}$$

$$\begin{array}{l} =\mathrm{Min}\left[\left(1–p\right)\alpha_{1},\;\mathrm{Min}\left\{\left(1–p\right)\alpha_{1}\alpha_{2},\;Cp\left(1–\beta_{1}\right)\left(1–\beta_{2}\right)\right\}+\mathrm{Min}\{\left(1–p\right)\alpha_{1}\left(1–\alpha_{2}\right),\;Cp\left(1–\beta_{1}\right)\beta_{2}\}\right]\\ \;+\mathrm{Min}\left[\left(1–p\right)\left(1–\alpha_{1}\right),\mathrm{Min}\left\{\left(1–p\right)\left(1–\alpha_{1}\right)\alpha_{2},\;Cp\beta_{1}\left(1–\beta_{2}\right)\right\}+\mathrm{Min}\left\{\left(1–p\right)\left(1–\alpha_{1}\right)\left(1–\alpha_{2}\right),Cp\beta_{1}\beta_{2}\right\}\right]. \end{array}$$

Now,

$$\left(1–p\right)\alpha_{1}=\mathrm{Min}\left[\left(1–p\right)\alpha_{1},\;\mathrm{Min}\left\{\left(1–p\right)\alpha_{1}\alpha_{2},\;Cp\left(1–\beta_{1}\right)\left(1–\beta_{2}\right)\right\}+\mathrm{Min}\left\{\left(1–p\right)\alpha_{1}\left(1–\alpha_{2}\right),Cp\left(1–\beta_{1}\right)\beta_{2}\right\}\right]$$

only if:

$$\begin{array}{l} \left(1–p\right)\alpha_{1}<\left(1–p\right)\alpha_{1}\alpha_{2}+\left(1–p\right)\alpha_{1}\left(1–\alpha_{2}\right)\\ 0<\left(1–p\right)\alpha_{1}\alpha_{2}+\left(1–p\right)\alpha_{1}–\left(1–p\right)\alpha_{1}\alpha_{2}–\left(1–p\right)\alpha_{1}\\ 0<0,\;\text{which is a contradiction}. \end{array}$$

And,

$$\left(1–p\right)\left(1–\alpha_{1}\right)=\mathrm{Min}\left[\left(1–p\right)\left(1–\alpha_{1}\right),\;\mathrm{Min}\left\{\left(1–p\right)\left(1–\alpha_{1}\right)\alpha_{2},Cp\beta_{1}\left(1–\beta_{2}\right)\right\}+\mathrm{Min}\left\{\left(1–p\right)\left(1–\alpha_{1}\right)\left(1–\alpha_{2}\right),\;Cp\beta_{1}\beta_{2}\right\}\right]$$

only if:

$$\begin{array}{l} \left(1–p\right)\left(1–\alpha_{1}\right)<\left(1–p\right)\left(1–\alpha_{1}\right)\alpha_{2}+\left(1–p\right)\left(1–\alpha_{1}\right)\left(1–\alpha_{2}\right)\\ 0<\left(1–p\right)\left(1–\alpha_{1}\right)\alpha_{2}+\left(1–p\right)\left(1–\alpha_{1}\right)–\left(1–p\right)\left(1–\alpha_{1}\right)\alpha_{2}–\left(1–p\right)\left(1–\alpha_{1}\right)\\ 0<0,\;\text{which is a contradiction}. \end{array}$$

Therefore,

$$\begin{array}{l} \mathrm{Min}\left[\left(1–p\right)\alpha_{1},\mathrm{Min}\left\{\left(1–p\right)\alpha_{1}\alpha_{2},Cp\left(1–\beta_{1}\right)\left(1–\beta_{2}\right)\right\}+\mathrm{Min}\{\left(1–p\right)\alpha_{1}\left(1–\alpha_{2}\right),Cp\left(1–\beta_{1}\right)\beta_{2}\}\right]\\ +\mathrm{Min}\left[\left(1–p\right)\left(1–\alpha_{1}\right),\;\mathrm{Min}\left\{\left(1–p\right)\left(1–\alpha_{1}\right)\alpha_{2},Cp\beta_{1}\left(1–\beta_{2}\right)\right\}+\mathrm{Min}\left\{\left(1–p\right)\left(1–\alpha_{1}\right)\left(1–\alpha_{2}\right),Cp\beta_{1}\beta_{2}\right\}\right]\\ =\mathrm{Min}\left\{\left(1–p\right)\alpha_{1}\alpha_{2},Cp\left(1–\beta_{1}\right)\left(1–\beta_{2}\right)\right\}+\mathrm{Min}\left\{\left(1–p\right)\alpha_{1}\left(1–\alpha_{2}\right),\;Cp\left(1–\beta_{1}\right)\beta_{2}\right\}\\ +\mathrm{Min}\left\{\left(1–p\right)\left(1–\alpha_{1}\right)\alpha_{2},Cp\beta_{1}\left(1–\beta_{2}\right)\right\}+\mathrm{Min}\left\{\left(1–p\right)\left(1–\alpha_{1}\right)\left(1–\alpha_{2}\right),Cp\beta_{1}\beta_{2}\right\} \end{array}$$

The last expression is identical to the expression for the expected cost for deciding on the response to two independent detectors after the results from both are known, as derived in Sec. 6. Since there is no incremental cost to getting the second detector’s report, the expected cost from using an IDS composed of two independent detectors is the same regardless of whether the response decision is made sequentially after each report or if it is made only once on the basis of both reports.

## 8. Appendix C. Simplification of the Decision Analysis and Geometry of ROC

### 8.1 Removal of Embedded Decision

Consider the decision tree shown in Fig. 2 and its expected cost: Min{*Cβp*, (1 − *α*)(1 − *p*)} + Min{*C*(1 − *β*)*p*, *α*(1 − *p*)}. The four possible combinations of expressions are the expected costs from following each of four strategies: never respond, always respond, follow the IDS’s recommendation (i.e., respond only to alarms), and do the opposite of what the IDS recommends (i.e., respond only to no alarm). Recognize that the strategies of always responding and never responding reduce to the strategy of following the IDS’s recommendation for an IDS operating at the points (1, 1) and (0, 0), respectively.

Thus, the decision tree in Fig. 2 can be simplified to remove the embedded decision if it can be shown that it is never optimal to do the opposite of the IDS’s recommendation. This is the case for a convex ROC curve (i.e., one where 1 − *β* > *α* for at least one combination of *α* and *β*). One would either follow the IDS’s recommendation or one would always respond or never respond. Assume that it is optimal to follow the IDS’s recommendation, then:

“Follow” is better than “Never Respond”: *Cp* > (1 − *p*) *α* + *Cpβ*;
“Follow” is better than “Always Respond”: 1 − *p* > (1 − *p*) *α* + *Cpβ*; and
“Follow” is better than “Opposite”: *Cp*(1 − *β*) + (1 − *p*)(1 − *α*) > (1 − *p*) *α* + *Cpβ*.

Now,

- *Cp* > (1 − *p*) *α* + *Cpβ* ⇒ *Cp*(1 − *β*) > [(1 − *p*) *α* + *Cpβ*](1 − *β*); and
- 1 − *p* > (1 − *p*) *α* + *Cpβ* ⇒ (1 − *p*)(1 − *α*) > [(1 − *p*) *α* + *Cpβ*](1 − *α*).

Thus,

$$\begin{array}{c} \left[\left(1–p\right)\alpha +Cp\beta \right]\left(1–\beta \right)+\left[\left(1–p\right)\alpha +Cp\beta \right]\left(1–\alpha \right)>\left(1–p\right)\alpha +Cp\beta \\ \Rightarrow Cp\left(1–\beta \right)+\left(1–p\right)\left(1–\alpha \right)>\left(1–p\right)\alpha +Cp\beta . \end{array}$$

Now,

$$\begin{array}{l} \left[\left(1–p\right)\alpha +Cp\beta \right]\left(1–\beta \right)+\left[\left(1–p\right)\alpha +Cp\beta \right]\left(1–\alpha \right)>\left(1–p\right)\alpha +Cp\beta \\ \text{if}:\left(1–\beta \right)+\left(1–\alpha \right)>1,\;\text{that is for}\;1–\beta >\alpha . \end{array}$$

However, if an IDS has a concave ROC curve (i.e., one where 1 − *β* < *α*), then cost could be minimized by doing the opposite of what the IDS recommends. It is important to notice that a concave ROC can be as valuable as a convex one. Any IDS with a ROC curve that differs from the ROC curve of no IDS, 1 − *β* = *α*, offers some information and is better than no IDS for some combination of cost and prior probability of intrusion. Doing the opposite of what the IDS recommends is optimal if:

“Opposite” is better than “Never Respond”: (1 − *p*)(1 − *α*) + *Cp*(1 − *β*) < *Cp*;
“Opposite” is better than “Always Respond”: (1 − *p*)(1 − *α*) + *Cp*(1 − *β*) < 1 − *p*; and
“Opposite” is better than “Follow”: (1 − *p*)(1 − *α*) + *Cp*(1 − *β*) < (1 − *p*) *α* + *Cpβ*.

Now,

iii. (1 − *p*)(1 − *α*) + *Cp*(1 − *β*) < *Cp* ⇒ [(1 − *p*)(1 − *α*) + *Cp*(1 − *β*)]*β* < *Cpβ*; and
iv. (1 − *p*)(1 − *α*) + *Cp*(1 − *β*) < 1 − *p* ⇒ [(1 − *p*)(1 − *α*) + *Cp*(1 − *β*)]*α* < (1 − *p*)*α*.

Thus,

$$\begin{array}{c} \left(1–p\right)\left(1–\alpha \right)+Cp\left(1–\beta \right)<\left[\left(1–p\right)\left(1–\alpha \right)+Cp\left(1–\beta \right)\right]\alpha +\left[\left(1–p\right)\left(1–\alpha \right)+Cp\left(1–\beta \right)\right]\beta \\ \Rightarrow \left(1–p\right)\left(1–\alpha \right)+Cp\left(1–\beta \right)<\left(1–p\right)\alpha +Cp\beta . \end{array}$$

Now,

$$\begin{array}{l} \left(1–p\right)\left(1–\alpha \right)+Cp\left(1–\beta \right)<\left[\left(1–p\right)\left(1–\alpha \right)+Cp\left(1–\beta \right)\right]\alpha +\left[\left(1–p\right)\left(1–\alpha \right)+Cp\left(1–\beta \right)\right]\beta \\ \text{if}\;1<\alpha +\beta ,\;\text{that is for}\;1–\beta <\alpha . \end{array}$$

Furthermore, a concave ROC curve with parameters *α* and *β* is equivalent to a convex ROC curve with parameters *α*′ and *β*′, where *α*′ = 1 − α and *β*′ = 1 − *β*. The expected cost from following the convex ROC with parameters *α* and *β* is equal to the expected cost from acting opposite to the concave ROC with parameters *α*′ and *β*′.

$$\begin{array}{l} \left(1–p\right)\alpha +Cp\beta =\left(1–p\right)\left(1–\alpha^{\prime }\right)+Cp\left(1–\beta^{\prime }\right)\text{if}\\ \left(1–p\right)\alpha +Cp\beta =\left(1–p\right)\left[1–\left(1–\alpha \right)\right]+Cp\left[1–\left(1–\beta \right)\right],\\ \left(1–p\right)\alpha +Cp\beta =\left(1–p\right)\alpha +Cp\beta . \end{array}$$

So, if the extreme points of (0, 0) and (1, 1) are considered part of the ROC curve and if points where 1 − *β* < *α* are transformed by setting *α*′ = 1 − α and *β*′ = 1 − *β*, then the embedded decision can be eliminated from the decision tree, and the expected cost is simply: *α*(1 − *p*) + *Cβp*. The structure of the simplified decision tree is shown in Fig. 16.

Summarizing, an IDS that includes the point (0, 1) is a perfect IDS. An IDS that includes the point (1, 0) is also a perfect IDS, since, by doing the opposite of its recommendation, one achieves performance equivalent to the IDS with the point (0, 1). An IDS that includes only the points (0, 0), (1, 1), or any along the straight line connecting these two, is a worthless non-IDS. All other IDSs are better than none but not as good as perfect.

### 8.2 ROC Convex Hull

Provost and Fawcett [15] describe a method that they call the ROC convex hull method for evaluating IDSs. Simply stated, the method builds a composite ROC curve from several ROC curves by taking the convex hull of all available curves. This identifies the ROC curves that are optimal for some cost and prior probability combinations. They do not recognize the value of concave ROC curves, but their method is trivially extended to include the convex equivalents of concave ROCs.

In the case of IDSs that offer sets of points for an ROC curve, the convex hull will be piecewise linear, and the determination of the optimal setting is the linear programming problem with the feasible region bounded by line segments connecting the points on the convex hull of ROC curves and the extreme points of (0, 0) and (1, 1), and the objective function is to minimize: α(1 − *p*) + *Cβp*.

The geometry of the situation is shown for a ROC curve with a single point in Fig. 14. The IDS is better than no IDS as long as the slope of the cost minimization objective function is between the slope of the line through (0, 0) and (*α*_0_, 1 − *β*_0_) and the slope of the line through (*α*_0_, 1 − *β*_0_) and (1, 1). This suggests several metrics for the ROC curve. A ROC curve is preferred over no curve over a greater range of objective functions if *θ* is smaller (0.5π < *θ* < π). Alternatively, a ROC curve is preferred over no curve over a greater range of objective functions if sin(*θ*) is larger (0 < sin(*θ*) < 1). This is consistent with the statement that a ROC curve is preferred over no curve over a greater range of objective functions if the area, *A*, under the ROC curve is larger (0 < *A* < 1). Since the area under the “no IDS” line is equal to 0.5, the metric *A*′ = *A* − 0.5 is probably a better metric than *A*. The angle *θ* can be determined from the coordinates of the ROC point as follows:

tan *θ* = (*m*_2_ − *m*_1_)/(1 + *m*_2_ *m*_1_), where *m*_1_ = (1 − *β*_0_)/*α*_0_ and *m*_2_ = *β*_0_/(1 − *α*_0_). Solving for *θ*, *θ* = tan^−1^ *α*_0_ *β*_0_ − (1 − *α*_0_) (1 − *β*_0_)]/[*α*_0_(1 − *α*_0_) + *β*_0_(1 − *β*_0_)]}. The area between the IDS’s ROC curve and the curve for no IDS is: *A*′ = 0.5 (1 − *β*_0_ − *α*_0_).

However, if anything is known about *p* or *C*, the ranges on these parameters over which the IDS is better than no IDS are probably better metrics than any of the four mentioned above. Operating at the point on the ROC curve is best if the following two conditions hold:

- *Cp* > (1 − *p*)*α*_0_ + *Cpβ*_0_ , and
- 1 − *p* > (1 − *p*)*α*_0_ + *Cpβ*_0_.

Solving for *C*:

$$\left[\left(1–p\right)/p\right)\left(\alpha_{0}/\left(1–\beta_{0}\right)\right]<C<\left[\left(1–p\right)/p\right]\left[\left(1–\alpha_{0}\right)/\beta_{0}\right].$$

Or, solving for *p*:

$$\alpha_{0}/\left[C\left(1–\beta_{0}\right)+\alpha_{0}\right]<p<\left(1–\alpha_{0}\right)/\left(1–\alpha_{0}+C\beta_{0}\right).$$

A similar result obtains for the case where the ROC curve is more than one point, which is illustrated in Fig. 15. Here, the IDS is better than none if:

$$\begin{array}{c} \left[\left(1-p\right)/p)(\alpha_{1}/\left(1-\beta_{1}\right)\right]<C<\left[\left(1-p\right)/p)(\left(1-\alpha_{2}\right)/\beta_{2}\right],\text{or},\;\text{equivalently},\;\text{if}:\\ \alpha_{1}/\left[C\left(1-\beta_{1}\right)+\alpha_{1}\right]<p<\left(1-\alpha_{2}\right)/\left(1-\alpha_{2}+C\beta_{2}\right). \end{array}$$

Here, the angle over which the IDS is better than no IDS is:

$$\theta =\tan^{-1}\left\{\left[\alpha_{1}\beta_{2}-\left(1-\alpha_{2}\right)\left(1-\beta_{1}\right)\right]/\left[\alpha_{1}\left(1-\alpha_{2}\right)+\beta_{2}\left(1-\beta_{1}\right)\right]\right\}.$$

### 8.3 Costs On All Outcomes

A similar analysis can be conducted for the general case where costs are associated with all four combinations of intrusion and response. For an analysis based on expected cost, the actual costs can be transformed by first subtracting the cost of one outcome from the costs of the others and then dividing all costs by one of the remaining non-zero costs. Now the costs of the outcomes are all expressed as a linear function of the original costs. Since the expectation of a linear function of a random variable is the same linear function of its expectation, these operations preserve the expected cost calculation.

This transformation results in the event tree for the case of general costs shown in Fig. 16. Costs of event combinations are scaled relative costs. They are relative to the cost of no response and no intrusion (the quiescent cost). They are scaled to units of the amount that the false alarm cost exceeds the quiescent cost. With this cost structure, the objective function is to minimize: *K*(1 − *β*)*p* + *α*(1 − *p*) + *Cβp*. There are now two cost ratios, *C* and *K*. Solving for the range over which the IDS is better than no IDS in terms of the prior probability, *p*, yields:

$$\alpha_{1}/\left[\alpha_{1}+\left(C–K\right)\left(1–\beta_{1}\right)\right]<p<\left(1–\alpha_{2}\right)/\left[1–\alpha_{2}+\left(C–K\right)\beta_{2}\right].$$

Let *D* = *C* − *K*, the difference in cost between failing to respond to an intrusion and responding to an intrusion. The usual condition is that *C* > *K* or, alternatively *D* > 0 (i.e., it is better to respond to an intrusion than to not respond). Solving for *D*, the range over which the IDS is better than no IDS in terms of this cost difference is:

$$\alpha_{1}\left(1–p\right)/\left[\left(1–\beta_{1}\right)p\right]<D<\left(1–\alpha_{2}\right)\left(1–p\right)/\beta_{2}p.$$

## 9. Appendix D. Composite ROC Curves

This appendix shows that a composite ROC curve can be obtained from two component ROC curves. The composite curve depends on the component ROC curves and the decision rule used to respond to alarms from either or both of the component IDSs. This analysis assumes that each component ROC curve is convex or the convex equivalent of a concave ROC curve (as described in Sec. 8). In this case, the decision analysis can be simplified to consider only the cases of responding to alarms. In general, one must consider the decision rules of: 1) responding only if both component IDSs indicate an alarm, 2) responding only if one of the components IDSs (labeled IDS 1) indicates an alarm, 3) responding only if the other component IDS (labeled IDS 2) indicates an alarm, or 4) responding if either IDS indicates an alarm.

In terms of the parameters of the IDSs, the prior probability of intrusion, *p*, and the cost ratio, *C*, described in Sec. 2, the expected costs from following the decision rules are as follows. The expected cost of responding to both alarms is:

$$\begin{array}{l} EC_{B}=\left(1–p\right)\alpha_{1}\alpha_{2}+Cp\left(1–\beta_{1}\right)\beta_{2}+Cp\beta_{1}\left(1–\beta_{2}\right)+Cp\beta_{1}\beta_{2}\\ \;=\left(1–p\right)\alpha_{1}\alpha_{2}+Cp\left(\beta_{1}+\beta_{2}–\beta_{1}\beta_{2}\right). \end{array}$$

The expected cost of responding to IDS 1’s alarms is:

$$\begin{array}{l} EC_{1}=\left(1–p\right)\alpha_{1}\alpha_{2}+\left(1–p\right)\alpha_{1}\left(1–\alpha_{2}\right)+Cp\beta_{1}\left(1–\beta_{2}\right)+Cp\beta_{1}\beta_{2}\\ \;=\left(1–p\right)\alpha_{1}+Cp\beta_{1}. \end{array}$$

The expected cost of responding to IDS 2’s alarms is:

$$\begin{array}{l} EC_{2}=\left(1-p\right)\alpha_{1}\alpha_{2}+Cp\left(1-\beta_{1}\right)\beta_{2}+\left(1-p\right)\left(1-\alpha_{1}\right)\alpha_{2}+Cp\beta_{1}\beta_{2}\\ \;=\left(1-p\right)\alpha_{2}+Cp\beta_{2}. \end{array}$$

The expected cost of responding to either alarm is:

$$\begin{array}{l} EC_{E}=\left(1-p\right)\alpha_{1}\alpha_{2}+\left(1-p\right)\alpha_{1}\left(1-\alpha_{2}\right)+\left(1-p\right)\left(1-\alpha_{1}\right)\alpha_{2}+Cp\beta_{1}\beta_{2}\\ \;=\left(1-p\right)\left(\alpha_{1}+\alpha_{2}-\alpha_{1}\alpha_{2}\right)+Cp\beta_{1}\beta_{2}. \end{array}$$

Notice that the cases where the decision rule is to respond to a single IDS’s alarm are equivalent to using the single IDS. Thus, the parts of the composite IDS’s ROC curve for the conditions under which the optimal decision rule is to respond to just a single IDS’s alarm is the same as that IDS’s ROC. For conditions where the other decision rules are optimal, the composite ROC will have effective values of *α* and *β* that can be calculated from the parameters of the component IDSs. Compare the expected value results above with the expected value equations for a single IDS as given in Sec. 8.1. Notice that the factor associated with (1 − *p*) is the effective value of *α*, and the factor associated with *Cp* is the effective *β*. Therefore, when the optimal decision rule is to respond only when both component IDSs indicate an alarm, the effective parameters of the composite ROC curve are: *α*_B_ = *α*_1_*α*_2_ and *β*_B_ = *β*_1_ + *β*_2_ − *β*_1_*β*_2_. When the optimal decision rule is to respond to an alarm from either component IDS, the effective parameters of the composite ROC curve are: *α*_E_ = *α*_1_ + *α*_2_ − *α*_1_*α*_2_ and *β*_E_ = *β*_1_*β*_2_.
